# Pro-inflammatory Macrophages Sustain Pyruvate Oxidation through Pyruvate Dehydrogenase for the Synthesis of Itaconate and to Enable Cytokine Expression*

**DOI:** 10.1074/jbc.M115.676817

**Published:** 2015-12-17

**Authors:** Johannes Meiser, Lisa Krämer, Sean C. Sapcariu, Nadia Battello, Jenny Ghelfi, Aymeric Fouquier D'Herouel, Alexander Skupin, Karsten Hiller

**Affiliations:** From the Luxembourg Centre for Systems Biomedicine, University of Luxembourg, 6 Avenue de Swing, L-4367 Belvaux, Luxembourg

**Keywords:** immunology, inflammation, macrophage, metabolic regulation, metabolism, mitochondrial metabolism, pyruvate;, Itaconate

## Abstract

Upon stimulation with Th1 cytokines or bacterial lipopolysaccharides, resting macrophages shift their phenotype toward a pro-inflammatory state as part of the innate immune response. LPS-activated macrophages undergo profound metabolic changes to adapt to these new physiological requirements. One key step to mediate this metabolic adaptation is the stabilization of HIF1α, which leads to increased glycolysis and lactate release, as well as decreased oxygen consumption. HIF1 abundance can result in the induction of the gene encoding pyruvate dehydrogenase kinase 1 (PDK1), which inhibits pyruvate dehydrogenase (PDH) via phosphorylation. Therefore, it has been speculated that pyruvate oxidation through PDH is decreased in pro-inflammatory macrophages. However, to answer this open question, an in-depth analysis of this metabolic branching point was so far lacking. In this work, we applied stable isotope-assisted metabolomics techniques and demonstrate that pyruvate oxidation is maintained in mature pro-inflammatory macrophages. Glucose-derived pyruvate is oxidized via PDH to generate citrate in the mitochondria. Citrate is used for the synthesis of the antimicrobial metabolite itaconate and for lipogenesis. An increased demand for these metabolites decreases citrate oxidation through the tricarboxylic acid cycle, whereas increased glutamine uptake serves to replenish the TCA cycle. Furthermore, we found that the PDH flux is maintained by unchanged PDK1 abundance, despite the presence of HIF1. By pharmacological intervention, we demonstrate that the PDH flux is an important node for M(LPS) macrophage activation. Therefore, PDH represents a metabolic intervention point that might become a research target for translational medicine to treat chronic inflammatory diseases.

## Introduction

Macrophages are innate immune cells that differentiate from monocytes, which circulate in the bloodstream. Upon differentiation, they invade the surrounding tissue and become resident macrophages (1). Macrophages can be activated by cytokines or toll-like receptor agonists, *e.g.* lipopolysaccharide (LPS). In very general terms, macrophage activation can result in rather pro-inflammatory responses, serving as host defense mechanisms or in wound healing responses and aiding in tissue repair and remodeling. However, depending on the type of activation, very different subtypes of activation occur (2). Upon activation with LPS (M(LPS)) or the cytokine interferon-γ (M(INFγ)), macrophages undergo profound metabolic reprogramming, necessary to activate cellular defense mechanisms as well as to cope with different micro-environments in the inflamed tissue (3). A marker for pro-inflammatory activation is high expression of *Irg1* (4, 5). We recently showed that *Irg1* codes for the enzyme *cis*-aconitate decarboxylase (IRG1/CAD) that catalyzes the synthesis of the antimicrobial metabolite itaconate from *cis*-aconitate (6). Therefore, metabolic reprogramming due to these adaptations could affect the availability of substrate needed for the synthesis of itaconate during host defense.

The reprogramming during macrophage activation shows overlapping features with cancer cells; both have increased glycolytic rates and increased lactate release, known as aerobic glycolysis or the Warburg effect (7, 8). Increased glycolytic rates are observed in any cell type that exceeds the energy demand derived from oxidative phosphorylation (9). In macrophages, it has been revealed that this metabolic rewiring is mediated by stabilization of HIF1α (10). A well described HIF1 target is the gene encoding pyruvate dehydrogenase kinase 1 (PDK1), an inhibitor of Pyruvate dehydrogenase (PDH)^3^ (11). When HIF1 is stabilized, the PDH flux can be inhibited by PDK1-mediated phosphorylation, resulting in decreased pyruvate-derived acetyl-CoA levels. In this case, reductive carboxylation of α-ketoglutarate (αKG) increases to provide sufficient acetyl-CoA for lipogenesis, needed for cell proliferation (12–15). Increased glycolytic rates, decreased PDH flux, and increased reductive carboxylation can also be induced in any cell type when oxygen tension is decreased or when oxidative phosphorylation is impaired. In this case, NADH oxidation is compromised, and citrate levels decrease. This decrease in citrate is linked to an increase in NADH, which thermodynamically promotes the metabolic adaptation to hypoxia by increasing reductive carboxylation of αKG (13, 16, 17). However, it is currently unclear whether this full panel of metabolic consequences upon HIF1 stabilization is also true for pro-inflammatory macrophages. Although it has been demonstrated that HIF1α can be stabilized and activated by cytokines, *e.g.* TNFα and IL1β (18, 19), the detailed metabolic consequences are still uncertain.

HIF1 can bind to the promoter of the *Tlr4* gene, thereby acting through a positive feedback loop on the TLR4 defense axis (20). Reactive oxygen species were shown to inhibit proteasomal degradation of HIF1α (21), and recently, increased succinate levels in pro-inflammatory macrophages have also been described to stabilize HIF1α, by inhibiting prolyl hydroxylase-mediated hydroxylation of HIF1α (10).

In this study, we asked whether the stabilization of HIF1α in M(LPS) macrophages alters TCA cycle fluxes similarly to hypoxic cells. We show that LPS-activated macrophages exhibit a metabolic adaptation with overlapping features to hypoxic cells, but also distinct differences, resulting in a unique LPS-specific metabolic signature. The most striking difference to hypoxic cells with stabilized HIF1α is that there is no decrease in relative glucose flux through PDH and no increase in reductive carboxylation of αKG. The sustained PDH flux provides sufficient acetyl-CoA for citrate production, which keeps the rate of reductive carboxylation of αKG at a low level, as well as providing citrate that is needed for the synthesis of fatty acids and itaconate. Upon LPS stimulation, high PDH activity is maintained by a repression of *Pdk1*. As a result PDH phosphorylation on Ser-232 and Ser-293 remains at a low level. Finally, we demonstrate that the PDH flux is indeed important to sustain M(LPS) activation in macrophages.

## Experimental Procedures

### 

#### 

##### Chemicals

UK5099 (Sigma, PZ0160) was first diluted in DMSO and further diluted in medium to a working stock concentration of 10 mm and then added to the wells of the culture dish to a final concentration of 100 μm. The final concentration of DMSO did not exceed 1%; LPS (*Escherichia coli,* Sigma L6529) was prepared as a working stock of 1 μg/ml in glucose- and glutamine-free DMEM and added 1:100 into the wells of the culture dish for a final concentration of 10 ng/ml. Cells were stimulated for 6 h; interferon γ (mouse) (*E. coli*, Sigma I4777) was prepared as a working stock of 5 mg/ml in glucose- and glutamine-free DMEM and added 1:100 into the wells of the culture dish for a final concentration of 50 ng/ml. Cells were stimulated for 6 h.

##### Stable Isotope Tracing

^13^C stable isotope tracers were obtained from Eurisotop as follows: glucose, CLM-1396; glutamine, CLM-1822-H. The tracer medium was prepared in DMEM without glucose and glutamine. [^13^C]Glucose (final 25 mm) and [^12^C]glutamine (final 4 mm) (or vice versa) was added to the medium; pH was set to 7.3; 10% FBS and 1% penicillin/streptomycin were added, and finally, the medium was sterile-filtered. Tracer medium was prepared in advance and incubated in the hypoxia chamber overnight to remove excessive oxygen from the medium (only for hypoxia conditions). The next morning, medium was replaced by tracer medium, and cells were incubated for 24 h with the tracer medium to reach isotopic steady state. LPS was added after 24 h and left on the cells for an additional 6 h prior to extraction. For further information regarding stable isotope-assisted metabolomics, see also Refs. 22, 23.

##### Cell Culture

RAW 264.7 cells (ATCC® TIB-71^TM^) were obtained from ATCC and were cultivated according to the manufacturer's instructions. Briefly, cells were kept in DMEM5796 with 10% FBS and 1% penicillin/streptomycin at 37 °C and 5% CO_2_ under atmospheric oxygen concentrations. In the case of hypoxia, cells were cultivated in an incubator at 37 °C and 5% CO_2_ located in a closed hypoxia chamber with an oxygen concentration of 2%. Cells were simultaneously seeded in the afternoon at a density of 150,000 cells per well in 12-well plates (Thermo Scientific, Nunc Multidish 12 Nunclon Delta Si) and then either incubated either at 21 or 2% O_2_ overnight. The next day, medium was replaced. In the case of hypoxia, medium was already incubated in the hypoxia chamber overnight to remove excessive oxygen from the medium. For comparison reasons, medium for normoxic conditions was also prepared the day before and incubated in a similar way under atmospheric oxygen conditions. For LPS activation, LPS was added 18 h after medium replacement. 24 h after medium replacement cells were harvested.

##### Viability Assay

To assess cell viability, cells were transferred into tubes and analyzed using a Vi-Cell^TM^ XR (Beckman Coulter) automated cell counter. To determine viability, cells were incubated with trypan blue and number of stained cells was determined.

##### cDNA Synthesis and Gene Expression Analysis

cDNA synthesis and quantitative PCR was performed as described previously (6). Briefly, RNA was isolated from the interphase after extraction of metabolites, using the Qiagen RNeasy mini kit. 0.5–1 μg of RNA was used for cDNA synthesis using SuperScript III (Invitrogen), following the manufacturer's instructions. Quantitative PCR was performed using iQ SYBR Green Supermix (Bio-Rad) as per the manufacturer's instructions. PCR was carried out on a Light Cycler 480 (Roche Applied Science). Data analysis was performed using the analysis software according to manufacturer's instructions (Roche Applied Science). Gene expression was normalized to the housekeeping gene *L27*. For primer sequences, see Table 1.

**TABLE 1:** **Primer sequences used for quantitative PCR**

Gene	5′ to 3′ sequence
*L27*_F	ACATTGACGATGGCACCTC
*L27*_R	GCTTGGCGATCTTCTTCTTG
*Irg1*_F	GCAACATGATGCTCAAGTCTG
*Irg1*_R	TGCTCCTCCGAATGATACCA
*Tnfa*_F	GGTTCTGTCCCTTTCACTCAC
*Tnfa*_R	TGCCTCTTCTGCCAGTTCC
*iNo*s_F	AGCCCTCACCTACTTCCTG
*iNos*_R	CAATCTCTGCCTATCCGTCTC
*Pdk1*_F	TGCAAAGTTGGTATATCCAAAGCC
*Pdk1*_R	ACCCCGAAGCTCTCCTTGTA
*Il1b*_F	TGCCACCTTTTGACAGTGATG
*Il1b*_R	TGATGTGCTGCTGCGAGATT
*Cpt1*_F	TGGCAGTCGACTCACCTTTC
*Cpt1*_R	CAAACAGTTCCACCTGCTGC
*Hif1a*_F	TGACGGCGACATGGTTTACA
*Hif1a*_R	AATATGGCCCGTGCAGTGAA

##### Extraction of Intracellular Metabolites

Extraction was according to Ref. 24. Briefly, cells were cultivated in 12-well plates and washed with 1 ml of 0.9% NaCl and quenched with 0.2 ml of −20 °C methanol. After adding an equal volume of 4 °C cold water, cells were collected with a cell scraper and transferred to tubes containing 0.2 ml −20 °C chloroform. The extracts were shaken at 1400 rpm for 20 min at 4 °C (Thermomixer Eppendorf) and centrifuged at 16,000 × *g* for 5 min at 4 °C. 0.2 ml of the upper aqueous phase was collected in specific glass vials with micro inserts and evaporated under vacuum at −4 °C using a refrigerated CentriVap Concentrator (Labconco).

##### Gas Chromatography-Mass Spectrometry

Metabolite derivatization was performed using a Gerstel MPS. Dried polar metabolites were dissolved in 15 μl of 2% methoxyamine hydrochloride in pyridine at 40 °C under shaking. After 60 min, an equal volume of MTBSTFA was added and held for 60 min at40 °C. 1 μl of sample was injected into an SSL injector at 270 °C in splitless mode. GC/MS analysis was performed using an Agilent 7890A GC equipped with a 30-m DB-35MS + 5-m Duraguard capillary column. Helium was used as carrier gas at a flow rate of 1.0 ml/min. The GC oven temperature was held at 100 °C for 2 min and increased to 300 °C at 10 °C/min. After 3 min, the temperature was increased to 325 °C. The GC was connected to an Agilent 5975C inert XL MSD, operating under electron ionization at 70 eV. The MS source was held at 230 °C and the quadrupole at 150 °C. The MS was operated in selected ion monitoring. The total run time of one sample was 25.00 min. All GC/MS chromatograms were processed by using Metabolite Detector software (25). MIDs were determined and corrected for natural isotope abundance using Metabolite Detector software.

Measurement of glucose and lactate intensities was performed by derivatization with an equal volume of MSTFA (instead of MTBSTFA) and held for 30 min at 40 °C under continuous shaking. A 1-μl sample was injected into an SSL injector at 270 °C in split 10 mode. GC oven temperature was held at 90 °C for 1 min and increased to 300 °C at 15 °C/min for 8 min to 320 °C. The total run time of one sample was 24.3 min. For absolute quantification of glucose and lactate, a dilution series of a standard mix was included in the sequence and measured in triplicate. For normalization, we used [U-^13^C]glucose and [U-^13^C]lactate as internal standards (in this case, medium samples contained only [^12^C]carbon sources).

##### Quantification of Amino Acids

Quantification of amino acids was performed on an Agilent 1100 HPLC system equipped with a Diode Array Detector. Separation was carried out on a ZORBAX amino acid analysis column (150 × 4.6 mm, 5 μm) with a preceding ZORBAX amino acid analysis guard cartridge (Agilent Technologies, Santa Clara, CA) at 40 °C in gradient mode (see Table 1). The eluents used were 40 mm Na_2_HPO_4_ (pH 7.8, eluent A) and a mixture of acetonitrile, methanol, and water (45:45:10, eluent B). 0.02% sodium azide was added to eluent A to prevent microbial growth. Primary amines were automatically derivatized with *ortho*-phthalaldehyde in borate buffer (0.4 n in water, pH 10.2) and diluted in eluent A prior to injection. The resulting *ortho*-phthalaldehyde derivatives were subsequently detected at 338 nm (10-nm bandwidth; reference wavelength, 390 nm; 20-nm bandwidth). All medium samples were diluted 1:1 with the internal standard l-2-aminobutyric acid (final concentration, 300 μm) to correct for deviations resulting from the derivatization process. External calibration standards as well as reference media with known concentrations were measured with every run to determine sample concentrations and ensure stability of the analysis. Gradient profile: 1.9 min, 0% eluent B; 18.1 min, 57% eluent B; 18.6 min, 100% eluent B; 22.3 min, 100% eluent B; 23.2 min,0% eluent B; 26 min, 0% eluent B.

##### Western Blot

For preparation of whole cell extract, 1 × 10^6^ cells were harvested, washed with ice-cold 1× phosphate-buffered saline (PBS) (Invitrogen/Life Technologies, Inc., Europe BV Belgium), lysed in 1× M-PER®, mammalian protein extraction Reagent (Thermo Scientific, Belgium) completed with 1× protease inhibitor mixture (Complete®, Roche Applied Science, Luxembourg), and further processed according to the manufacturer's instructions. A nanodrop analyzer was used to measure the protein concentration. Proteins were separated by size using SDS-PAGE (12%) and transferred to an Immobilon-FL PVDF membrane (Merck Millipore) using the Mini-PROTEAN Tetra Cell and PowerPac Basic Power Supply (Bio-Rad, Belgium). The membrane was blocked in 5% nonfat milk powder in PBS/Tween for 1 h at room temperature or overnight at 4 °C. The antibodies used were as follows: anti-IRG1 (Sigma, hpa040143) 1:750 in PBS-T 5% nonfat milk powder for 1 h at room temperature; anti-PDK1 (rabbit) (Enzo catalog no. ADI-KAP-PK112) 1:3000 in TBST 5% BSA, overnight at 4 °C; anti-PDH-E1α (rabbit) (Ser(P)-232) (Millipore catalog no. AP1063) 1:1000 in TBST 5% BSA, overnight at 4 °C; anti-PDH-E1α (rabbit) (Ser(P)-300) (Millipore catalog no. ABS194) 1:1000 in TBST 5% BSA, overnight at 4 °C; anti-PDH-E1α (rabbit) (Ser(P)-293) (Millipore catalog no. ABS204) 1:10,000 in TBST 5% BSA, overnight at 4 °C; anti-α-tubulin (mouse) 1:5000 in TBST 5% BSA, overnight at 4 °C; anti-rabbit HRP 1:5000 in TBST 5% skim milk (Cell Signaling); anti-mouse HRP 1:5000 in TBST 5% skim milk (Cell Signaling). Visualization was done using the ECL Plus Western blotting detection system Kit (GE Healthcare, Netherlands). Signals were detected using the LI-COR system. Quantification of band intensities was done using the ImageStudioLight Software package.

##### Cell Imaging and Data Analysis

Phase contrast images were acquired using a ×10 objective on a Nikon Ti Eclipse inverted microscope with motorized stage (Nikon Corp., Tokyo, Japan) enclosed in a bench top incubator. Automatic microscope control, stage programming, and acquisition were done using the OptoMorph version of MetaMorph 7.8.10 (Cairn Research, Kent, UK). LPS-treated and untreated RAW 264.7 cells growing in 12-well Nunc plates (50,000 cells per well) were imaged in positive phase contrast. Nine adjacent but non-overlapping images were automatically acquired in a 3 × 3 grid around the center of each well. The entire imaging experiment was performed twice, resulting in 36 images acquired per condition. One out-of-focus image and five containing cell aggregates were excluded from further analysis. Surfaces of strongly attached cells were estimated by thresholding in ImageJ 2.0.0-rc-31/1.49v using the IJ_IsoData algorithm. Congruence of thresholding results with cell contours was verified by visual inspection. Cell sizes were calculated using the particle analysis tool, allowing for areas from 50 to 5000 pixels and excluding particles on the image edges. Average sizes were reported for each image. Halo and contrast effects resulting from the phase-contrast imaging were analyzed to discriminate between strongly and weakly attached cells. Strongly attached cells appeared darker than background with a weak halo, although weakly attached cells appeared brighter than background with a strong halo. Histograms of pixel intensities of the entire field of view were analyzed using custom software written in MATLAB R2014b. First, the intensity peak corresponding to the background signal (cell-free surface) was identified and approximated by a Gaussian function. The approximated peak was subtracted from the original histogram, and the weighted sum of intensities below (dark) and above (bright) the background peak was calculated, respectively. The ratio of dark-to-bright values informs on the ratio of strongly-to-weakly attached cells. Renormalization of this ratio by values obtained for individual strongly and weakly attached cells yielded an adhesion index with values from 0 (no cells attached) to 1 (all cells attached).

##### Statistical Analyses

To analyze a significant difference between two groups, unpaired Welch's *t* test was applied. *p* values are indicated in each panel (*, *p* < 0.01; or as indicated) and was considered as significantly different with a *p* value <0.05. The number of independent replicates is indicated in the figure legends. Each experiment was at least performed three times with cells of a different passage. Each individual experiment consisted of three wells per condition.

## Results

### 

#### 

##### M(LPS) Macrophages Show a Distinct Metabolite Signature

The macrophage environment is likely to be hypoxic, as these cells infiltrate hypoxic tissues such as tumors, wounded regions, or sites of inflammation. At the site of inflammation, different oxygen tensions result from increased oxygen demand as well as swelling or vascular damage (26, 27). To physiologically meet these challenging conditions, macrophages are well adapted to hypoxia by their ability to adjust their gene expression profile and increase glycolytic activity (28). To study whether the activation of macrophages reprograms metabolism to a hypoxia-like phenotype, we cultivated the murine macrophage cell line RAW 264.7 under normoxia (21% oxygen) and hypoxia (2%) and activated it with LPS for 6 h. We selected this time point, because it reflects the highest expression level of *irg1/Cad*, the enzyme that catalyzes the synthesis of the antimicrobial metabolite itaconate (6).

**FIGURE 1.**
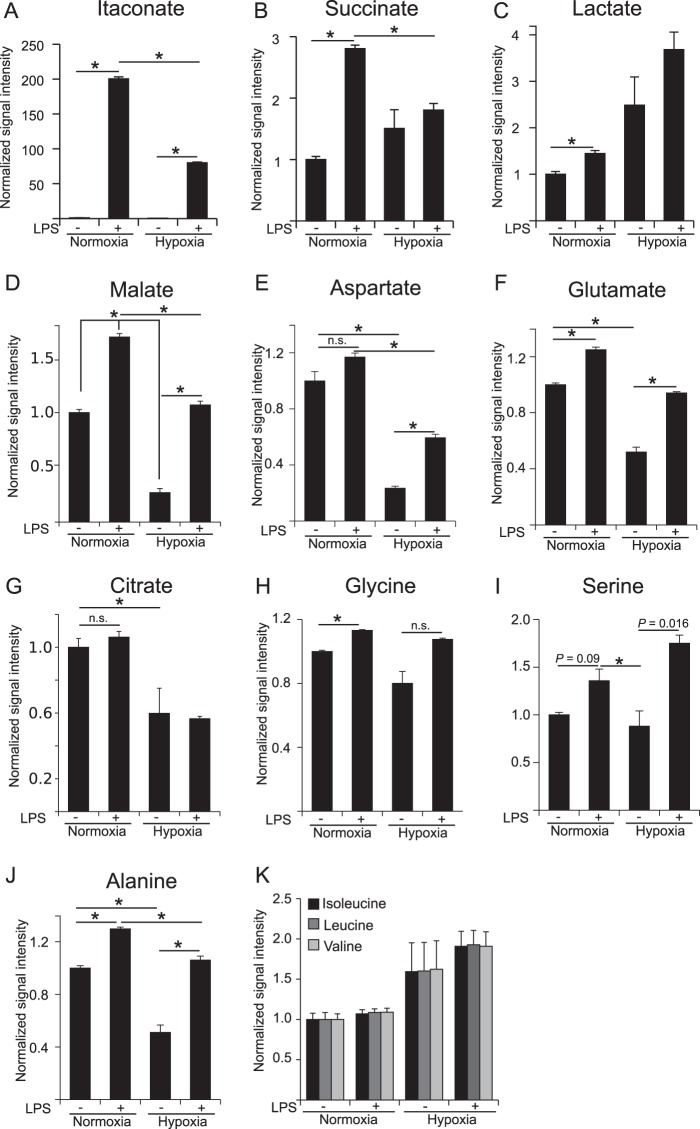
**Metabolome analysis of RAW 264 macrophages in the context of LPS activation and hypoxia.** Analysis of intracellular metabolite abundance in RAW 264 cells, cultivated under normoxia (21% O_2_) and hypoxia (2% O_2_), unstimulated or stimulated with LPS. Metabolites were extracted and analyzed by GC/MS. Signal intensities (peak area) are normalized to unstimulated cells under normoxia. Cells were treated with 10 ng/ml LPS for 6 h. *Error bars* indicate S.E. (Welsh's *t* test; *, *p* < 0.01, *n* = 3 wells). *n.s.,* not statistically significant. One representative experiment with three individual wells per condition is presented. The experiment was performed three times.

We analyzed intracellular metabolite levels and revealed that LPS stimulation resulted in increased levels of itaconate, succinate, and lactate under normoxia (Fig. 1, *A–C*). These three metabolites are marker metabolites for M(LPS) activation, demonstrating a clear pro-inflammatory activation (6, 10, 29, 30). Moreover, we observed increased levels of the TCA cycle associated metabolites malate and glutamate, whereas aspartate and citrate levels remained unchanged (Fig. 1, *D–G*). The amino acids glycine, serine, and alanine were also elevated upon LPS stimulation (Fig. 1, *H–J*). Compared with normoxia, LPS treatment of hypoxic cells was unable to induce similar itaconate and succinate levels (Fig. 1, *A* and *B*). As expected, lactate levels further increased upon hypoxia as a result of reduced respiration and concomitant increased glycolysis, known as the Pasteur Effect. Non-activated hypoxic cells exhibited decreased levels of the TCA cycle associated metabolites malate, aspartate, glutamate, and citrate. However, LPS stimulation under hypoxia resulted in increased levels of malate, aspartate, and glutamate but not citrate (Fig. 1, *D–G*). The amino acids glycine and serine were unchanged between hypoxia and normoxia. Upon LPS activation of hypoxic cells, serine and alanine were increased (Fig. 1, *H–J*). Interestingly, we also observed a trend of increased levels of the branched chain amino acids isoleucine, leucine, and valine under hypoxia for both non-stimulated and LPS stimulated conditions (Fig. 1*K*; *p* value >0.05).

We concluded from these results that LPS affects the metabolite profile of macrophages independent of the oxygen supply and that this profile is clearly different from a pure hypoxic profile. Intriguingly, we observed decreased abundance of the antimicrobial metabolite itaconate under oxygen-limiting conditions, which could have direct effects on the immune response in tissues with low oxygen tension.

##### Decreased Itaconate Levels under Hypoxia Are Not the Result of Decreased Irg1 Expression or Lower IRG1/CAD Protein Abundance

**FIGURE 2.**
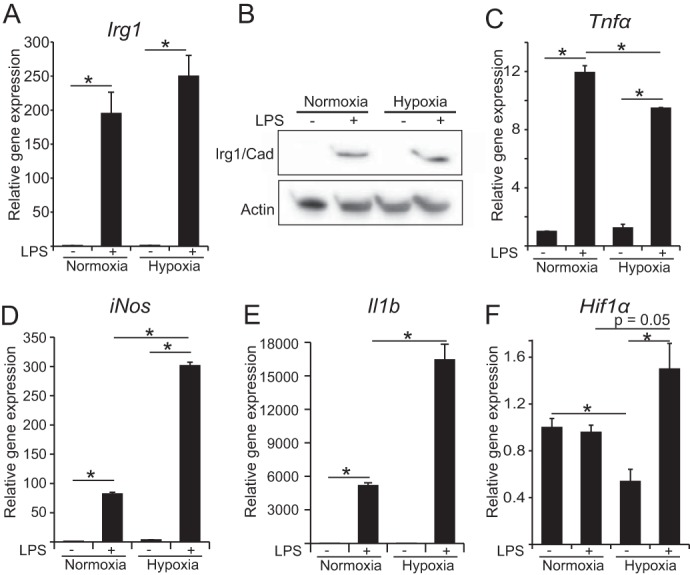
**Expression analysis of pro-inflammatory associated genes.** *A,* relative gene expression of *Irg1. B,* Western blot against IRG1/CAD, the protein that catalyzes the conversion of *cis*-aconitate to itaconate (no significant difference (data not shown)). *C–F,* relative gene expression of *Tnf*α, *iNos, Il1*β, and *Hif1*α, normalized to normoxia control (−*LPS*). *Error bars* indicate S.E. (Welsh's *t* test, *, *p* < 0.01, *n* = 3). Gene expression data represents the mean over three independent experiments.

To investigate whether the reduced levels of itaconate are caused by transcriptional repression, post-transcriptional regulation, or post-translational regulation, we analyzed gene expression and protein levels of the catalyzing enzyme IRG1/CAD. We found that hypoxia does not significantly reduce the gene expression level or the protein abundance of IRG1/CAD, indicating a regulation at the post-translational level or a result of decreased substrate concentrations (Fig. 2, *A* and *B*). To confirm LPS activation of macrophages, we analyzed gene expression of the pro-inflammatory marker genes *Tnf*α, *iNos,* and *Il1*β (Fig. 2, *C–E*). We found clear up-regulation under both oxygen conditions when the cells were treated with LPS. However, hypoxia resulted in a 20% reduction of *Tnf*α expression and ∼3-fold higher expression of *Il1*β and *iNos*.

It has been reported in several recent publications that HIF1α gets stabilized in M(LPS) macrophages compared with resting macrophages (10, 21, 29, 31). Therefore, we also analyzed gene expression of *Hif1*α, and we observed increased expression of *Hif1*α in response to LPS treatment under hypoxia (Fig. 2*F*), indicating that HIF1α stabilization in hypoxic M(LPS) macrophages can be a combined effect of increased transcriptional expression and post-translational stabilization. Under normoxia, no difference in *Hif1*α expression was detected.

Based on these results, we conclude that the reduction of itaconate under hypoxia is mostly a result of post-translational effects such as altered metabolic fluxes, either due to an enzyme modification that regulates its activity or by changing substrate concentrations. To that end, we were interested to compare cellular flux changes with LPS activation between hypoxia and normoxia.

##### LPS Activation Promotes Pyruvate Oxidation via PDH by Preventing Pdk1 Expression

**FIGURE 3.**
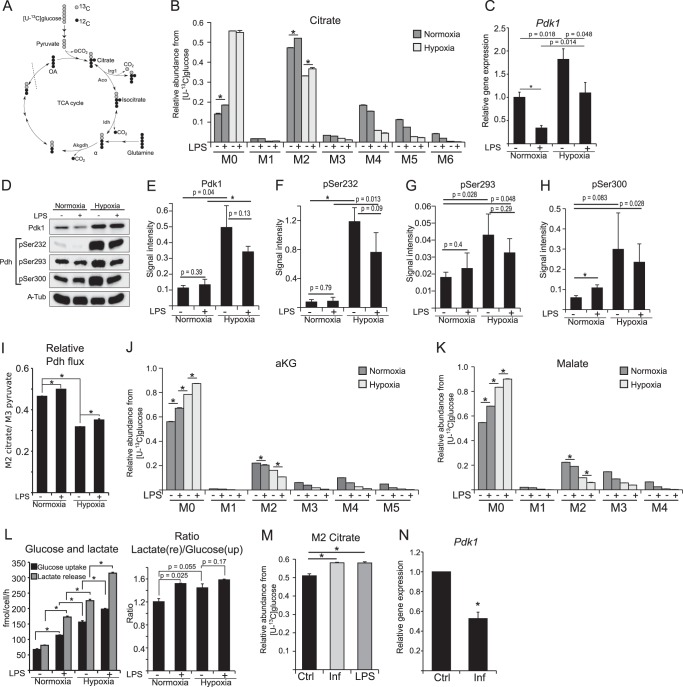
**PDH flux analysis in M(LPS) macrophages.** *A,* schematic of atom transitions in central metabolism using [U-^13^C]glucose as a tracer for determination of MIDs to infer relative intracellular fluxes. ^13^C-carbons are in *gray* and ^12^C in *black*. The *dotted line* indicates end of one route. *Aco,* aconitase; *Idh,* isocitrate dehydrogenase; *Akgdh,* αKG dehydrogenase; *OA,* oxaloacetate. *B,* MID of citrate. *M0* to *M6* indicates the different mass isotopologues. *C,* gene expression analysis of *Pdk1. D–H,* Western blot analysis of PDK1 and PDH phosphorylation on Ser-232, -293, and -300. α-Tubulin serves as loading control. *D* shows one representative Western blot of three independent experiments. α*-Tub*, α-tubulin. *E–H* shows quantification of three independent experiments. *I,* ratio of M2 citrate/M3 pyruvate indicating relative pyruvate oxidation through PDH. *J* and *K,* MID of αKG and malate. *L,* absolute quantification of glucose uptake and lactate release, and ratio of lactate release/glucose uptake to infer fractional lactate formation per glucose. *M,* activation of RAW 264 cells with 10 ng/ml LPS or 50 ng/ml interferon-γ (*INF*) in medium with [U-^13^C]glucose. Presented are citrate M2 isotopologues as a readout for relative glucose flux through PDH. *N,* relative gene expression of *Pdk1* normalized to normoxia control (*Ctrl*) (−*LPS*). *Error bars* indicate S.E. (Welsh's *t* test, *, *p* < 0.01, *n* = *3*). One representative experiment with three individual wells per condition is presented. Each experiment was performed at least three times, except for *C.* Presented is the mean ± S.E. over three independent experiments. (Welsh's *t* test, *, *p* < 0.01, *n* = 3).

To monitor intracellular glucose-derived fluxes in M(LPS) macrophages, we incubated RAW 264 cells for 24 h in the presence of uniformly labeled [U-^13^C]glucose to reach isotopic steady state conditions. During these 24 h, labeled glucose is metabolized within the cells and is incorporated in cellular metabolites downstream of glucose. To obtain the specific labeling patterns, intracellular metabolites were extracted and analyzed with gas chromatography/mass spectrometry to determine MIDs (corrected for natural isotope abundance), which reflect relative metabolic fluxes (Fig. 3*A* for atom transitions) (22, 23).

As expected, stable isotope labeling revealed that hypoxia resulted in decreased pyruvate flux through PDH, illustrated by decreased M2 citrate isotopologues (Fig. 3*B*). Concomitant with decreasing citrate M2 isotopologues under hypoxia, we observed increased abundance of citrate M0 isotopologues, representing citrate molecules derived from carbon sources other than glucose (Fig. 3*B*). This decrease is a result of HIF1α stabilization and *Pdk1* induction, where the PDK1 protein inhibits PDH by phosphorylation (32, 33). Decreased pyruvate oxidation under hypoxia promotes decreasing downstream substrate levels, most probably resulting in the observed decreased itaconate levels (Fig. 1*A*). However, HIF1α has also been shown to be stabilized in M(LPS) macrophages under normoxic conditions (10, 29). Therefore, it is speculated that pyruvate oxidation through PDH is decreased in M(LPS) macrophages, a similar phenotype to cancer cells with stabilized HIF1α (3, 34). However, we did not observe decreased citrate M2 isotopologues in normoxic M(LPS) macrophages, raising the question whether PDH is inhibited under these conditions.

To analyze regulation of *Pdk1* in more detail, we investigated gene expression and protein abundance of PDK1 and phosphorylation status of PDH (Fig. 3, *C–H*). In line with current knowledge, hypoxia-mediated stabilization of HIF1α resulted in increased *Pdk1* expression (Fig. 3*C*), which complements our observation of hypoxia-dependent, decreased glucose flux to citrate (Fig. 3*B*). Intriguingly, we observed that LPS stimulation resulted in a drastic reduction of *Pdk1* expression at both normoxia and hypoxia, indicating that PDK1-mediated inhibition of PDH is reduced in M(LPS) macrophages (Fig. 3*C*). Moreover, analysis of PDK1 protein abundance revealed similar PDK1 levels in non-activated or M(LPS)-activated macrophages. Only in hypoxia did we observe a significant increase of PDK1 abundance (Fig. 3, *D* and *E*). To investigate whether the low levels of PDK1 have an effect on PDH, we analyzed phosphorylation of PDH on serine 232, 293, and 300 (Fig. 3, *D* and *F–H*). Phosphorylation of these residues has been reported to result in an inhibition of PDH activity (33). As expected, hypoxia resulted in increased phosphorylation of the three analyzed serine residues (not significant in case of Ser-300). However, and in line with low PDK1 levels in normoxic M(LPS) macrophages, LPS activation did not increase phosphorylation levels of Ser-232 and -293. Regarding Ser-300, we observed significantly increased phosphorylation upon LPS activation, although there was no further significantly increased phosphorylation due to hypoxia. Whether Ser-300 specifically has a unique function in the context of LPS activation in macrophages remains to be determined in future work. Our results from this part of the study reveal that HIF1-mediated induction of *Pdk1* can be attenuated by LPS stimulation, which is in line with increased citrate M2 isotopologues upon LPS activation (Fig. 3*B*).

In line with the results of PDK1 abundance and PDH phosphorylation status, our stable isotope analysis revealed that LPS stimulation did not result in a decreased relative pyruvate oxidation, both under hypoxia and normoxia (Fig. 3, *B* and *I*), indicating that HIF1 did not mediate PDH inhibition upon LPS stimulation. We observed increased citrate M2 isotopologue levels, reflecting even higher relative pyruvate flux through PDH (Fig. 3*B*). To normalize for upstream changes in glycolysis, we determined the ratio of M2 citrate/M3 pyruvate and observed an increase upon LPS stimulation, indicating increased pyruvate flux through PDH in M(LPS) macrophages (Fig. 3*I*). As a reduction of citrate M2 isotopologues was only observed under hypoxic conditions, HIF1-mediated inhibition of PDH depended only on the oxygen tension and not on the macrophage activation status. Indeed, LPS activation can even partially recover the HIF1- mediated PDH inhibition under hypoxia (Fig. 3, *B* and *I*).

The M2 abundance of αKG and malate was significantly lower than in citrate (Fig. 3, *J* and *K*), suggesting that large amounts of citrate, *cis*-aconitate, or isocitrate are either used for pathways other than the oxidative TCA cycle or that other carbon sources (*e.g.* glutamine) increase their contribution to αKG and malate. Stimulation with LPS as well as hypoxia further decreased glucose contribution to αKG and malate, as can be seen by a decreasing abundance of αKG and malate M2 isotopologues (Fig. 3, *J* and *K*).

Next, we analyzed glucose uptake from the medium and cellular lactate release, to evaluate whether M(LPS) macrophages increase their glycolytic rate. Under normoxia, LPS-stimulated macrophages increased glucose uptake and lactate release, and under hypoxia, glucose uptake and lactate release were exceeding the rate of M(LPS) macrophages under normoxia (Fig. 3*L*). This effect was further enhanced when hypoxic cells were additionally stimulated with LPS (Fig. 3*L*). Under normoxia, the lactate to glucose ratio was increased from 1.2 to 1.6 upon LPS stimulation, indicating increased activity of lactate dehydrogenase, which is in line with the Warburg-like phenotype of M(LPS) macrophages (3). However, 20% of the overall glucose carbon pool is still available for other metabolic pathways, indicating that M(LPS) macrophages still contain sufficient pyruvate to maintain oxidation through PDH.

It has been shown before that STAT3-specific signaling can inhibit PDH and thus block pyruvate oxidation through PDH in primary fibroblasts and cancer cell lines (35). Therefore, we investigated whether, and in contrast to LPS treatment, a STAT3-dependent activation with the cytokine INFγ might inhibit PDH. To this end, we treated the cells with INFγ or LPS and analyzed the isotope enrichment in citrate. We did not observe a difference between LPS and INFγ treatment (Fig. 3*M*). In both cases, M2 citrate was significantly increased compared with untreated controls. In line with LPS activation, we also observed decreased expression of *Pdk1* in M(INFγ) macrophages (Fig. 3*N*), indicating no inhibition of pyruvate oxidation in M(INFγ) macrophages. In conclusion, sustained pyruvate flux through PDH and its regulation by PDK1 seems to be independent of TLR4 signaling.

##### LPS Activation Increases Glutamine Uptake but Does Not Induce Reductive Carboxylation of αKG

**FIGURE 4.**
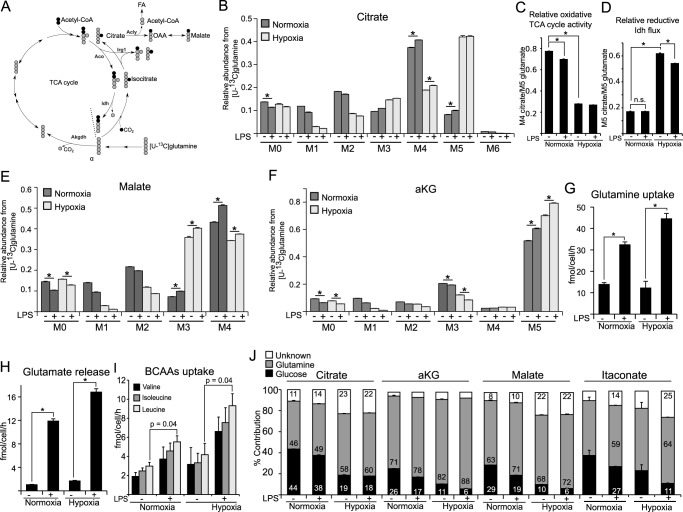
**Contribution of glutamine to central metabolism in M(LPS) macrophages.** *A,* schematic of atom transitions in central metabolism using [U-^13^C]glutamine as a tracer. ^13^C-carbons are in *gray*, ^12^C in *black*. Citrate molecules derived from reductive carboxylation of αKG are M5 isotopologues, whereas citrate molecules from the oxidative route of the TCA cycle are M4 isotopologues. The *dotted line* indicates end of one route. *Aco,* aconitase; *Idh,* isocitrate dehydrogenase; *Akgdh,* αKG dehydrogenase; *Acly,* ATP-dependent citrate lyase; *FA,* fatty acid. *B,* MID of citrate. *M0* to *M6* indicates the different mass isotopologues. *C* and *D,* determination of oxidative TCA cycle activity: ratio of M4 citrate/M5 glutamate and ratio of M5 citrate/M5 glutamate indicating relative reductive Idh flux. *E* and *F,* MID of malate and αKG. *G–I,* absolute quantification of glutamine uptake, glutamate release, and uptake of the branched chain amino acids (valine, isoleucine, and leucine). *J,* carbon contribution (%) of glucose, glutamine, and other carbon sources to citrate, αKG, malate, and itaconate. Carbon contributions are based on MIDs from [U-^13^C]glucose labeling (Fig. 3) and [U-^13^C]glutamine labeling. Carbon contribution to itaconate under non-LPS conditions should not be considered because itaconate levels are negligibly low under these non-stimulated conditions (Fig. 1*A*). *Error bars* indicate S.E. (Welsh's *t* test, *, *p* < 0.01, *n* = 3). One representative experiment with three individual wells per condition is presented. Each experiment was performed at least three times.

It has been demonstrated that hypoxic cells metabolize increased amounts of glutamine via reverse IDH activity to generate citrate by reductive carboxylation of αKG (12–15). This pathway fuels the citrate pool to provide sufficient acetyl-CoA for lipogenesis. Because M(LPS) activation also results in a stabilization of HIF1α (10, 19, 21, 29) and increased glycolytic flux (see Fig. 3, *I* and *L*), we investigated whether these cells exhibit increased reductive carboxylation of αKG. To that end, we applied a uniformly labeled [U-^13^C]glutamine tracer and determined MIDs of TCA cycle metabolites (Fig. 4*A* for atom transitions).

As expected, under hypoxic conditions we observed a drop in oxidative TCA cycle activity and a strong induction of reductive carboxylation, inferred from decreased citrate M4 and increased citrate M5 isotopologues (Fig. 4, *B–D*). This pattern was also reflected by decreasing malate M4 (oxidative route) and increasing malate M3 (reductive route) isotopologues (Fig. 4*E*). However, when cells were stimulated with LPS at normoxic conditions, reductive carboxylation of αKG was not increased (Fig. 4*D*).

Using the glutamine tracer, we observed that the major carbon source of αKG was glutamine and that upon LPS stimulation and under hypoxia the abundance of M5 isotopologues increased, suggesting an increase in glutamine influx and a decreasing glucose contribution (Fig. 4*F*). Although hypoxia results in increased usage of αKG for reductive carboxylation and decreased αKG oxidation (14, 15), we did not find evidence that LPS stimulation under normoxia compromises the relative glutamine carbon flux through αKG dehydrogenase, indicated by a similar enrichment pattern of αKG and malate.

To analyze glutamine metabolism in more detail, we quantified glutamine uptake from the medium and glutamate release from the cells. Although hypoxia did not result in increased glutamine uptake, we observed a strong increase of glutamine uptake upon LPS stimulation under both oxygen levels (Fig. 4*G*). Because glutamate is produced from glutamine and can be released from the cell, we quantified glutamate release to infer glutamine anaplerosis to the TCA cycle. Although we observed increased glutamate release upon LPS stimulation, the net uptake of glutamine in LPS-stimulated cells was still higher compared with untreated controls (control 12.84 *versus* LPS 20.5 fmol/cell/h) (Fig. 4, *G* and *H*). As indicated by the intracellular metabolite levels (Fig. 1*K*), we also observed a trend of increased consumption of branched chain amino acids from the medium upon LPS stimulation (Fig. 4*I*). However, these differences were only significant in the case of leucine. Nevertheless, we believe that the influence of branched chain amino acids on central metabolism is higher upon LPS activation.

To better understand the impact of the different carbon sources to the TCA cycle metabolites, we calculated the carbon contributions of glucose and glutamine to citrate, αKG, malate, and itaconate (Fig. 4*J*). We observed a small decrease in glucose contribution to citrate upon LPS activation. However, based on the [U-^13^C]glucose-derived MIDs, this decrease mostly originated from decreased citrate cycling through the oxidative TCA cycle (compare M3, M4, and M5 isotopologues in Fig. 3*B*) or increased glutamine influx, rather than decreased pyruvate flux through PDH (M2 isotopologue). Hypoxia resulted in a stronger reduction of glucose contribution to citrate, which was a result of PDH inhibition. Along with the decrease in glucose contribution, the glutamine contribution to citrate was increased upon LPS, especially under hypoxia, where glutamine is the major carbon source of citrate. For αKG and malate, the glucose contribution was significantly lower compared with citrate, indicating that major parts of the citrate pool are not used for further oxidation through the oxidative TCA cycle, but for anabolic processes such as lipid synthesis or itaconate synthesis. In line with this observation, we observed higher glucose contribution to itaconate, compared with αKG and malate. Moreover, upon LPS stimulation, and especially with hypoxia, we observed an increase of other carbon sources than glucose or glutamine, which is in line with potentially increased uptake of branched chain amino acids (Figs. 1*K* and 4*I*).

In summary, we observed that glutamine is the major carbon source of the TCA cycle in M(LPS) macrophages and is the main substrate for TCA oxidation. Glucose carbon still enters the cycle at citrate; however, significant amounts of the citrate pool are not further oxidized through IDH but are distributed to other metabolic pathways. Glutamine serves to replenish this lack of carbon by increased uptake upon LPS stimulation.

##### LPS Causes an Increase in Lipogenesis in RAW 264 Cells

**FIGURE 5.**
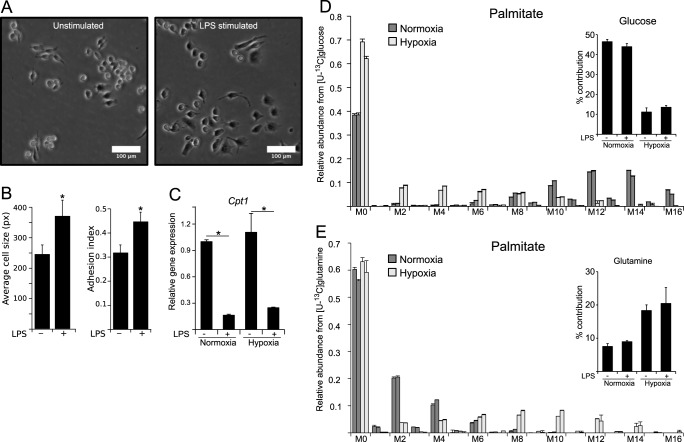
**Morphological changes upon LPS activation require sustained lipogenesis in macrophages.** *A,* microscopy (bright field image) of RAW macrophages unstimulated or stimulated with 10 ng/ml LPS for 6 h. *White bar* indicates 100 μm. *B,* mean average of cell size (pixels) (*left*) and analysis of adhesion index (*right*) of LPS-stimulated and -non-stimulated cells obtained from bright field microscopy. Analysis demonstrates morphological adaption of macrophages during LPS activation. For further details regarding the analysis approach, see under “Experimental Procedures.” *C,* relative gene expression of carnitine palmitoyl transferase1 (*Cpt1*) normalized to normoxia control (*Ctrl*) (−*LPS*), indicating inhibition of β-oxidation upon LPS stimulation. *D* and *E,* MID of palmitate using [U-^13^C]glucose (*D*) and [U-^13^C]glutamine (*E*) as a tracer. Contribution of each carbon source is depicted in the *top right corner* of each panel. *Error bars* indicate S.E. (Welsh's *t* test, *, *p* < 0.01, *n* ≥ 3). One representative experiment with three individual wells per condition is presented. Each experiment was performed at least three times, except for *C*. Presented is the mean ± S.E. over three independent experiments. (Welsh's *t* test, *, *p* < 0.01, *n* = 3).

Besides transcriptional and metabolic adaptations to inflammation, it has been described that pro-inflammatory macrophages also undergo morphological changes from small and spherical to a larger and more attached form (Fig. 5*A*). To demonstrate the surface enlargement during LPS activation, we monitored cell size by using two orthogonal microscopy approaches (Fig. 5*B*), demonstrating an increase in cell surface and thus pointing to an increased demand on lipids needed for membrane formation. Furthermore, M(LPS) macrophages increase intracellular and extracellular vesicle formation during pathogen defense (36). Therefore, M(LPS) macrophages have an increased demand for lipids, needed for morphological changes and vesicle formation. To meet this demand, LPS-activated macrophages need to prioritize their metabolism toward lipogenesis and to repress lipid oxidation. In line with this necessary metabolic shift, we observed decreased expression of carnitine palmitoyltransferase 1 (*Cpt1*), the gene of the protein that imports palmitate into the mitochondrion for lipid oxidation and subsequent energy production (Fig. 5*C*). A reduction of β-oxidation is in line with reports of decreased oxidative metabolism in M(LPS) macrophages (37) and supports the observed increased demand for lipids. High activity of lipid degradation, simultaneously to their synthesis, would be a waste of energy.

To generate lipids from central metabolism, cells export mitochondrial citrate into the cytoplasm where it is hydrolyzed via ATP-dependent citrate lyase to provide acetyl-coA for lipogenesis. In this preferred case, citrate utilization is for anabolic processes rather than the TCA cycle. To analyze the contribution of glucose and glutamine to palmitate, an end point of fatty acid synthesis, we determined the MIDs of and the carbon contributions to palmitate after both the application of [U-^13^C]glucose and [U-^13^C]glutamine as tracers in independent experiments (Fig. 5, *D* and *E*). We observed that under normoxia, nearly 50% of the carbon in palmitate originated from glucose. Under hypoxic conditions, a shift in isotopologues indicated that the glucose contribution was reduced while glutamine contribution was increased, suggesting increased reductive carboxylation under hypoxia, but not as a result of LPS stimulation. Although dependent on the pool sizes of the metabolites, increased production of itaconate and palmitate from glucose-derived citrate in M(LPS) macrophages indicates reduced citrate oxidation through the TCA cycle in M(LPS) macrophages.

##### Pyruvate Flux to Citrate Is Important for LPS Activation in RAW 264 Cells

**FIGURE 6.**
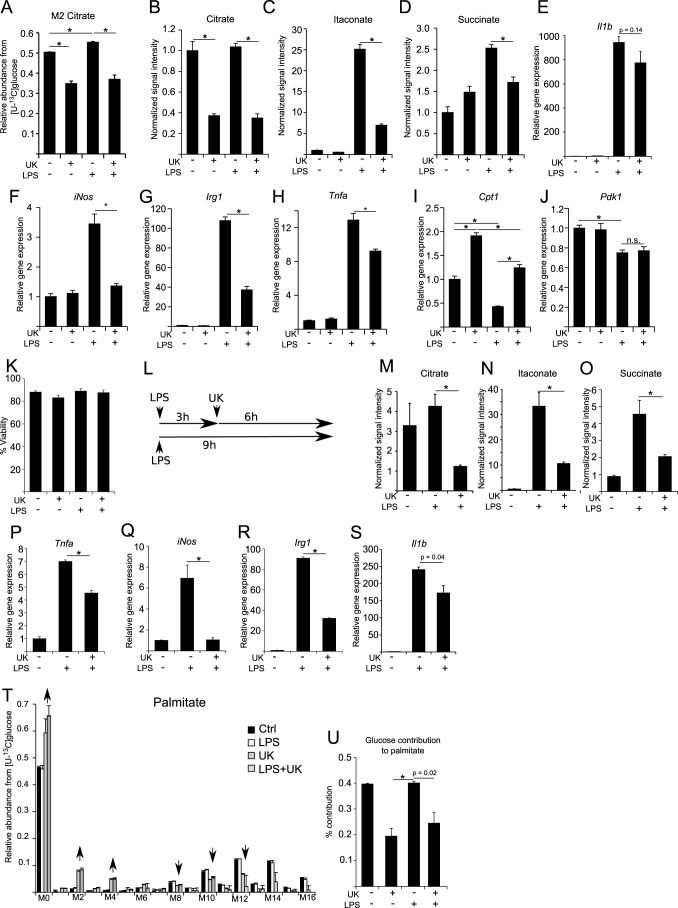
**Inhibition of pyruvate transport into mitochondria suppresses pro-inflammatory responses in M(LPS) macrophages.** *A,* application of the specific pyruvate transport inhibitor UK5099 to inhibit flux through PDH. Cells were treated with 100 μm UK5099 for 6 h with or without 10 ng/ml LPS at normoxia. Prior to treatment start, cells were cultivated for 24 h in [U-^13^C]glucose. Presented are citrate M2 isotopologues as a readout for relative glucose flux through PDH. *B–D,* intracellular metabolite levels of citrate, itaconate, and succinate upon LPS stimulation and after application of 100 μm UK5099. Metabolite levels were determined using GC/MS and normalized to cell number. *E–J,* relative gene expression of *Il1*β, *iNos, Irg1, Tnf*α, *Cpt1,* and *Pdk1* normalized to normoxia control (*Ctrl*) (−*LPS*), upon LPS stimulation and after application of 100 μm UK5099 for 6 h. *K,* viability assay to test a potential effect of UK5099 on cell viability. Assay was performed using trypan blue, dead and live cells were counted. *L,* to validate the effect of UK5099 a modified experimental setup has additionally been performed. Cells were first activated with 10 ng/ml LPS for 3 h and then UK5099 was added to the cells for additional 6 h. In total, LPS activation was 9 h in this case. Non-UK5099 treated and non-activated cells served as control. *M–O,* intracellular metabolite levels for citrate, itaconate, and succinate (analysis as in *B–D*). *P–S,* relative gene expression analysis of *Tnf*α, *iNos, Irg1,* and *Il1*β. *T* and *U,* MID of palmitate using [U-^13^C]glucose as a tracer. Experimental setup as in *A. U,* glucose contribution to palmitate, determined from *T. Error bars* indicate S.E. (Welsh's *t* test, *, *p* < 0.01, *n* = 3). One representative experiment with three individual wells per condition is presented. Each experiment was performed at least three times.

To investigate the importance of the PDH flux in M(LPS) macrophages, we used a pharmacological approach to impair pyruvate oxidation through PDH by inhibiting the pyruvate transporter with the specific inhibitor UK5099 (38). Using this inhibitor together with the [U-^13^C]glucose tracer, we observed a significant decrease of citrate M2 isotopologues, indicating reduced relative pyruvate oxidation through PDH (Fig. 6*A*). Addition of LPS to UK509-treated cells could not restore the abundance of M2 citrate, because pyruvate supply is impaired by the inhibition of the transporter. Analysis of intracellular metabolite levels revealed that inhibition of pyruvate transport into the mitochondrion resulted in decreased amounts of citrate, originating from reduced substrate levels for citrate synthase (Fig. 6*B*). Intriguingly, we also observed decreased amounts of the inflammatory marker metabolites itaconate and succinate under pro-inflammatory conditions (Fig. 6, *C* and *D*). Decreased amounts of itaconate and succinate could either result from decreased glucose flux into the TCA cycle, similar to hypoxia, or from a metabolic alteration in M(LPS) macrophages. To analyze these hypotheses, we investigated the gene expression profiles of pro-inflammatory marker genes (Fig. 6, *E–H*). Although *Il1b* gene expression was not significantly decreased (Fig. 6*E*), we observed that UK5099 treatment of LPS-stimulated macrophages resulted in decreased expression levels of *iNos*, *Irg1*, and *Tnf*α (Fig. 6, *F–H*), indicating that in this case and in contrast to hypoxia, the observed decreased itaconate level roots back to decreased *Irg1* levels. Moreover, application of UK5099 to LPS-stimulated cells resulted in increased *Cpt1* expression levels, similar to untreated controls, although *Pdk1* expression was still reduced, due to the fact that pyruvate import into the mitochondrion was inhibited and thus regulation of PDH not necessary (Fig. 6, *I* and *J*). To exclude a potential toxic effect of UK5099 on RAW 264 cells, we performed a viability assay and did not observe significant changes in viability (Fig. 6*K*). To further exclude the possibility of timing effects due to simultaneous addition of LPS and UK5099, we repeated the experiments with a modified experimental setup, where we first activated the cells with LPS for 3 h and then added UK5099 for an additional 6 h to investigate whether this intervention can repress the pro-inflammatory profile of M(LPS) macrophages (Fig. 6*L*). Following this approach, we also observed significantly decreased citrate, itaconate, and succinate levels (Fig. 6, *M–O*) and significantly decreased expression of *Tnf*α, *iNos*, *Irg1*, and *Il1b* (Fig. 6, *P–S*). These results indicate that inhibition of pyruvate import into the mitochondrion can indeed suppress pro-inflammatory responses in M(LPS) macrophages. Finally, we investigated the effect of carbon contribution to palmitate for these conditions, using [U-^13^C]glucose as a tracer, in combination with UK5099. In line with decreased citrate, itaconate, and succinate levels upon UK5099 treatment, we also observed lower carbon contribution from glucose to palmitate (Fig. 6, *T* and *U*).

In summary, these data demonstrate that the pyruvate flux through PDH is important for LPS activation in macrophages. Apparently, altered metabolic fluxes or changes in metabolite concentrations enable a feedback mechanism to regulate cellular gene expression profiles. Hence, metabolic intervention can be used to attenuate activation of M(LPS) macrophages.

## Discussion

M(LPS) macrophages require extensive reprogramming to enable host defense mechanisms. Although M(IL4) macrophages increase oxidative metabolism optimized for tissue repair (39), M(LPS) macrophages develop a Warburg-like phenotype by increasing glycolysis and lactate release (3). It has been demonstrated that pro-inflammatory activation results in the stabilization of HIF1α, HIF1 complex formation with monomeric or dimeric PKM2, along with an increased expression of M(LPS)-associated cytokines and bacterial defense mechanisms (29). While under hypoxia HIF1 not only increases the glycolytic activity but also represses PDH activity through PDK1, it was speculated that glucose flux through PDH is also repressed in M(LPS) macrophages (3, 40). However, this would diminish the carbon supply necessary for lipogenesis and synthesis of the antimicrobial metabolite itaconate.

In this work, we demonstrate that the PDH flux plays an important role in maintaining full LPS-specific activation and that pharmacological intervention to prevent pyruvate oxidation represses pro-inflammatory activation. Although an active PDH flux has been speculated for early macrophage differentiation and for dendritic cells before (3, 41), our results demonstrate that pyruvate oxidation through PDH is fully active in mature M(LPS) macrophages and that this is even essential to sustain LPS activation. Moreover, we discovered that this process is facilitated by repressed *Pdk1* expression and no increase in PDK1 protein abundance, as well as significantly lower phosphorylation of PDH compared with hypoxic conditions, illustrating that M(LPS) macrophages sustain an active pyruvate flux through PDH.

Under hypoxia, HIF1 decreases pyruvate oxidation through PDH by inducing *Pdk1* (32). In contrast to hypoxia, we demonstrated that although glycolysis is increased and oxygen consumption is decreased (29) in M(LPS) macrophages, pyruvate oxidation through PDH is not inhibited, because PDK1 abundance is not increased. Active PDH facilitates a stable citrate pool, which in turn prevents increased reductive carboxylation of αKG by thermodynamic means (16). M(LPS) macrophages have been shown to increase their expression of the mitochondrial citrate carrier to transport citrate from the mitochondrion into the cytosol (42), and we have shown that M(LPS) macrophages have an increased lipid demand. Because we demonstrated that reductive carboxylation of αKG is not increased, the high demand of citrate has to be mostly supplied by glucose through PDH.

When PDH flux is repressed, citrate is increasingly generated by reductive carboxylation of αKG, which is mostly derived from glutamine (43). An increased demand on glutamine in M(LPS) macrophages has been reported before (44). Here, we describe in detail how this glutamine is utilized. In M(LPS) macrophages most glutamine is preferentially used for glutaminolysis rather than for reductive carboxylation of αKG. Besides ATP-dependent citrate lyase-derived oxaloacetate, increased glutaminolysis additionally provides oxaloacetate, which is needed as an acceptor for pyruvate-derived acetyl-CoA and the synthesis of citrate. It also replenishes the TCA cycle to compensate for increased lipogenesis and the loss of carbon that is used for the synthesis of itaconate.

It is known that LPS activation results in decreased oxygen consumption, which suggests that TCA cycle activity is decreased and would provide less NADH to be oxidized via the ETC (29, 45, 46). However, we found evidence that the oxidative TCA cycle is still running and is fueled by increased amounts of glutamine. TCA cycle-derived NADH can be used for alternative routes and would consequently not be oxidized via the ETC and thus would also result in reduced oxygen consumption rates. Mitochondrially derived NADH can alternatively be converted to NADPH by nicotinamide nucleotide transhydrogenase (17, 47), which is utilized by M(LPS) macrophages for ROS production via NADPH oxidase, needed for antibacterial activity (48), or transferred to the cytoplasm (49) where it is needed for lipogenesis. Therefore, it is possible that M(LPS) macrophages use increased amounts of TCA cycle-derived NADH for the generation of NADPH rather than oxidizing it via the ETC, which fits to the reported decreased oxygen consumption rates (6, 45, 46). Additionally, the ETC can be directly inhibited by nitrosylation (50, 51) promoted by increased expression of *iNos*, which we have shown to be increased upon LPS stimulation. Therefore, decreased oxygen consumption in M(LPS) macrophages due to decreased ETC activity is reasonable, but a decreased ETC activity does not necessarily have to result in decreased oxidative TCA cycle activity.

Although we could demonstrate that PDH activity is important in M(LPS) macrophages, the mechanism of how *Pdk1* repression is facilitated despite the presence of HIF1 needs additional research. One possibility would be a weaker stabilization because HIF1α is not as abundant as in hypoxic cells. However, in this case, we would still expect a similar mode of action and therefore a moderate induction of *Pdk1*. LPS activation exhibits distinct regulatory modules (2), and we conclude that these specific networks overlap with the classical HIF1 signature to mediate LPS-specific activation. Because we demonstrated that M(LPS) macrophages depend on the PDH flux to induce cytokine expression and synthesis of itaconate, it appears that a specific HIF1-induced upstream regulator exists which specifically prevents *Pdk1* expression. In this way, increased glucose uptake and lactate release can still be facilitated by HIF1, whereas PDK1 activity is prevented by an additional regulator. Overall, this points toward a context-dependent HIF1 response.

With the hypoxia model, we demonstrated that the decreased pyruvate flux through PDH results in decreased levels of the antimicrobial metabolite itaconate as well as decreased levels of succinate. However, gene expression of *Irg1* was unchanged. We conclude that, under hypoxia, decreased metabolite levels are a passive effect of decreased pyruvate oxidation and decreased oxidative mitochondrial metabolism. This also illustrates that HIF1-mediated inhibition of PDH would be disadvantageous to M(LPS) macrophages and that parts of the TCA cycle are indeed important for M(LPS) macrophages, *e.g.* for the synthesis of lipids and itaconate.

By inhibiting the pyruvate import into the mitochondria of M(LPS) macrophages, we observed decreased metabolite levels of itaconate and succinate, similar to hypoxia. Intriguingly, in the case of transport inhibition, gene expressions of *Irg1, Tnf*α, and *iNos* were decreased as well. All of these genes are hallmarks of LPS activation, indicating a general impairment of activation in matured M(LPS) macrophages.

**FIGURE 7.**
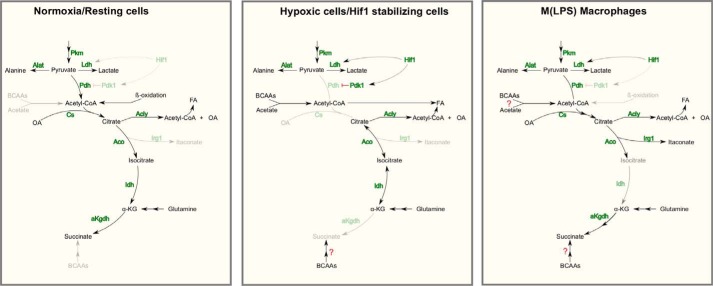
**Model summarizing the metabolic adaptations in M(LPS) macrophages.** Resting cells use glucose via PDH and TCA oxidation for energy production. Depending on the cell type, anabolic processes run in parallel. Hypoxic cells and cancer cells with stabilized HIF1α increase their glycolytic rate, inhibit PDH flux, and use glutamine-derived carbon for increased reductive carboxylation of αKG for subsequent lipogenesis. Highly proliferating cells promote anabolic processes to support proliferation. M(LPS) macrophages show stabilized HIF1α but do not decrease pyruvate oxidation through PDH. Under this condition, Hif1α does not increase PDK1 abundance. Compared with resting cells, less citrate is oxidized through the TCA cycle and is rerouted to serve as a precursor for itaconate and fatty acid synthesis. Glutamine serves to replenish the TCA cycle by increasing its carbon contribution to αKG and subsequent metabolites. BCAAs might also support carbon supply to the TCA cycle, but this needs additional research. Despite sustained pyruvate oxidation through PDH, oxygen consumption rates are lower than in resting cells.

Based on our observations, we can now extend the existing metabolic model of M(LPS) macrophages (Fig. 7). 1) We confirmed the findings of increased glycolysis and increased lactate release. 2) Furthermore, reductive carboxylation of αKG is not increased in M(LPS) macrophages, but instead glutamine is still oxidized through αKG dehydrogenase. 3) Finally, pyruvate is still oxidized through PDH, which turned out to be essential for full LPS activation. Our work revealed an LPS-specific metabolic feature that is essential for activation and that might be approached by further studies in the light of translational medicine to develop novel therapies against inflammatory dysfunctions.

## Author Contributions

J. M. designed the concept of this work, performed experiments, analyzed data, and wrote the manuscript. L. K. performed experiments and analyzed data. S. C. S. performed experiments, analyzed data, and revised the manuscript. N. B. and J. G. performed Western blots. A. F. and A. S. did the microscopy analysis. K. H. discussed experiments and revised the manuscript. All authors read and agreed to the content of this manuscript.

## Footnotes

3 The abbreviations used are:

PDH: pyruvate dehydrogenase
αKG: α-ketoglutarate
MIDS: mass isotopomer distribution.
